# Knowledge and Perceptions about Clinical Trials and the Use of Biomedical Samples: Findings from a Qualitative Study in Rural Northern Ghana

**DOI:** 10.1371/journal.pone.0152854

**Published:** 2016-04-01

**Authors:** Samuel Chatio, Frank Baiden, Fabian Sebastian Achana, Abraham Oduro, James Akazili

**Affiliations:** 1 Navrongo Health Research Centre, P.O Box 114, Navrongo, Ghana; 2 Ensign College of Public Health, Kpong, ER, Ghana; Renal Division, Peking University First Hospital, CHINA

## Abstract

**Introduction:**

Clinical trials conducted in sub-Saharan Africa have helped to address the prevalent health challenges. The knowledge about how communities perceive clinical trials is however only now evolving. This study was conducted among parents whose children participated in past clinical trials in northern Ghana to assess their knowledge and perceptions of clinical trials and the use of biomedical samples.

**Method:**

This was a qualitative study based on eighty in-depth interviews with parents. The participants were randomly selected from among parents whose children were enrolled in a clinical trial conducted in the Kassena-Nankana districts between 2000 and 2003. The interviews were transcribed and coded into emergent themes using Nvivo 9 software. The thematic analysis framework was used to analyze the data.

**Results:**

Study participants reported that clinical trials were carried out to determine the efficacy of drugs and to make sure that these drugs were suitable for human beings to use. The conduct of clinical trials was perceived to have helped to reduce the occurrence of diseases such as malaria, cerebrospinal meningitis and diarrhea. Quality of care was reported to be better in clinical trials than in the routine care. Parents indicated that participation in clinical trials positively influenced their health-seeking behavior. Apprehensions about blood draw and the use to which samples were put were expressed, with suspicion by a few participants that researchers sold blood samples. The issue of blood draw was most contentious.

**Conclusion:**

Parents perception about the conduct of clinical trials in the study districts is generally positive. However, misconceptions made about the use of blood samples in this study must be taken seriously and strategies found to improve transparency and greater community acceptability.

## Introduction

Randomized clinical trials (RCTs) are an important part of the process of evaluating the efficacy of new clinical interventions. Information from clinical trials help patients, clinicians, policy-makers and funding agencies to make informed clinical and health policy decisions [1, 2]. Clinical trials conducted in sub-Saharan Africa during the last decade have helped to address priority health problems such as malaria, tuberculosis, HIV and other neglected diseases in the region [3].

Reports on how clinical trials have influence the communities in which they are conducted is mixed. There is emerging concern that the impact of clinical trials on communities needs to be more rigorously evaluated [4]. In many places, there is the perception that the conduct of clinical trials either directly or indirectly impacted on the quality of routine health services [5, 6]. The conduct of clinical trials has also been reported to contribute to a mal-distribution of critically-needed health personnel, and provision of laboratory and other facilities in rural communities [5, 7]. Evidence exist that there is lack of trust by community members on the conduct of clinical trials [8]. Some community members put negative connotations on clinical trials as experiments. This is reported to discourage participation [9].

There is little evidence for assessing the impact of clinical trials activities on communities where these trials take place especially people who are recruited into these trials. Clinical trials in resource-poor settings could negatively impact the quality of health care, by diverting resources from routine clinical activities, by inducing “local-brain-drain” among the best qualified health staff and also the issues of discrimination among enrolled as against non-enrolled patients [9]. They may also have a negative impact on the relationships between the health facilities and the community due to a lack of knowledge of local culture and customs by trial researchers [10]. There is very little information on knowledge and perception of people on the conduct of clinical trials. Conversely, the presence of clinical trials and associated rumors regarding their “experimental” nature can adversely affect the acceptability and the full involvement of community members in the conduct of these trial studies [9]. The misconception on the use of blood samples during trial studies can have negative effect on the conduct of these trials [11, 12]. With the increase in the number of clinical trials conducted in Sub-Saharan Africa has come the need to examine these challenges more closely.

Vitamin A supplementation trial was the first clinical trial carried out by the Navrongo Health Research Centre in the study districts. This trial assessed the impact on childhood morbidity and mortality in northern Ghana [13]. A number of other clinical trials have been conducted in the district since then. This study was conducted among parents whose children participated in past clinical trials in northern Ghana to assess their knowledge and perceptions of the conduct of clinical trials and the use of biomedical samples.

## Methodology

### Ethical Considerations

Ethical approval for the study was received from the Navrongo Health Research Centre ethics committee (approval number NHRCIRB146). Written informed consent was obtained from each participant prior to being interviewed. The moderators of the interviews read and translated the consent forms into the local language preferred by the participants. All participants were informed about the purpose and procedure of the study, their right to refuse or withdraw from the study and the confidentiality of the information collected.

### Study Site

The study was conducted in the Kassena-Nankana East and West Districts (KNEWDs) by the Navrongo Health Research Centre (NHRC). The districts share borders with Burkina Faso in the north and covers an area of about 1675 km^2^. The districts have a combined population approximately 152,000 that is covered within the Navrongo Health Demographic Surveillance System (NHDSS) [14]. The centre was established in 1989 with the main aim of conducting high quality demographic and health research to inform policy. The centre has over the years conducted many community-based researches in the two Kassena-Nankana districts. The districts have two distinct seasons, a wet season that runs from May to September and a long dry season from October to April with hardly any rains. The main languages spoken in the two districts are Kasem and Nankani. The districts are predominantly rural with subsistence farming as the mainstay by the people.

The two districts have five health centers, two clinics, and 27 functional Community-based Health Planning and Services (CHPS) compounds located in various villages with resident Community Health Officers (CHOs) who offer doorstep reproductive health care services and also treat minor health conditions [15, 16]. The main referral hospital (War Memorial Hospital) is located at the capital (Navrongo Town) of the KNED. There are two pharmacy shops and over 50 drug and chemical shops located in various parts of the districts.

## Sampling of respondents

The participants in this study were randomly selected from among parents whose children were enrolled in a clinical trial conducted in the districts between 2000 and 2003 (ISRCTN46343627). The trial was conducted to assess the efficacy of using rectal artesunate as pre-referral medication [17]. A little over 2000 children were recruited into the study. Using the database of the list of participants in the trial, we used simple random sampling to select 120 parents for the interviews. This sampling technique was used to select participants in this study because there existed a database of the parents of children enrolled in the rectal artesunate trial. This approach was also sought to minimize subjective views expressed since some of the parents were known to the research staff and could be influenced into giving opinions based on their familiarity with the staff.

Since this trial ended in 2003 and a number of other trials have taken place in the district that targeted children in the same age band, it is conceivable that the participants sampled in this study may have had experience from other trials. We therefore anticipated that their views on clinical trials as expressed in this study could not be solely attributed to their experience during the index trial.

## Data collection procedure

The In-depth interviews were conducted by university graduates who had field experience in the conduct of qualitative research in the study area. Potential respondents in this study were contacted and consented. Arrangements were made with participants on a convenient date, place and time for the interviews to be conducted within the community. Each interview took between 40 to 50 minutes. Depending on the expressed preference of the respondents, interviews were conducted in Kassem, Nankani or English. A total of 80 parents whose children were recruited into the artesunate trial and possibly other clinical trials were interviewed. All of them consented to be part of the study.

### Data Processing and Analysis

We utilized the principle of saturation for data collection in this study. Data saturation was reached where no new or additional information is being found from the interviews [18]. Data collection, management and analysis were done concurrently. All interviews were audio-recorded and later transcribed verbatim after repeatedly listening to the recordings. The transcripts were then uploaded onto QSR Nvivo 9 software to facilitate data management and coding [19]. To ensure a fair interpretation of the data, the transcripts were initially coded by two researchers independently. Guided by the objectives of the study, the coding process involved a critical review (line-by-line) of each transcript to identify emerging themes from the data. The two coders then met to compare their independently-identified themes. They revolved divergence by re-reading the relevant sections of the transcripts together, and agreed on the best fit interpretation of the data. The major and sub-themes are discussed below, supported by relevant quotes from the transcripts.

## Results

### Background Information of Respondents

In all, 80 parents were interviewed in this study. The ages of the parents were grouped into three categories (24–34, 35–44 and 45–54 years). Most of them (forty two), were between 35–44 years, twenty-three were 45–54 years while fifteen of the parents were between 24–34 years. Most of them (seventy-five) were females while only five were males. The majority (forty-one) of them never had formal education. Only eight had at least secondary/tertiary education while thirty-one went up to primary/Junior high level. In terms of their occupation, thirty-nine of them were subsistence farmers, twenty-seven were traders, nine were civil/public servants and five of them were housewife/unemployed.

### Knowledge and Perceptions on the Conduct of Clinical Trials

Most of the parents reported that researchers needed to conduct clinical trial studies to test the drugs in order to be sure that the drugs were safe. Such trial studies needed to be carried out to determine the efficacy of these drugs and to come out with new knowledge on whether the drugs were suitable for human beings to use before they could be made available to a large number of people. They said this was to help prevent the harm that these drugs could cause to people if they were made available without testing. This was typically put as follows:

*“They have to do a study to find out about the potency of the drug whether it can cure people diseases or not*. *They cannot just start giving it to people without first testing it*, *people may die*. *That is why they have to do a study to see whether it is good before they can give it to many people to use”* ***(IDI- 37 year old Mother)***

Study respondents were of the view that even traditional medicines are usually tested on few selected people before it is used to treat many people. They concluded that it was normal for researchers to test modern drugs before allowing many people to use them.

*“…even in the local way if you find a new herb for treating a disease*, *you have to try it on yourself first or you select some people and test to know whether this herb is good or not*. *If you know that the herb is good because you have used it to treat someone and it works*, *I then take it (the herb) outside and safe the lives of those who are suffering from similar diseases” (****IDI-52 year old mother)*.**

### Drawing and Use of Biomedical Samples (Blood, Urine, Saliva and Stool)

Most of the respondents held the view that it was necessary for researchers to draw blood samples from trials participants because this enabled the researches know the exact disease or germ present in the blood. This they said helped researchers to treat study participants appropriately. Parents were of the view that it was not for fun that they had to take other samples such as urine, saliva and stool because some diseases might not be found in the blood but rather in saliva, stool or urine.

*“There is no problem with taking of blood samples… because when they do the test*, *they will be able to know the kind of diseases that the person has before they give the person treatment and so for that one I don’t see it to be a problem at all”* ***(IDI-35 year old Mother)*.**

*“[Laughter]*, *the reason is that there are certain diseases that are in the person’s urine while other diseases are in the blood and so when they take these samples and the disease is in the blood they will know*, *if it is in the urine they will know or if it is in the stool they will know and I think it is good for them to take those things and test”* ***(IDI-41 year old Mother)*.**

## Perceived Benefits of Clinical Trials

### Disease Reduction

Mosts of the parents reported that in the past, there used to be many “strange diseases” such as measles and convulsion that killed people especially children. According to them, they believed it was due to the activities of research centre that most of these diseases had been “eliminated”. They believed that the conduct of clinical trials had had the effect of reducing the incidence or effects of these diseases.

*“there used to be measles and convulsion; with the coming of NHRC workers and their activities*, *there is no more measles*, *no more convulsion*, *no more “zunzure” (caterpillar)*. *When it all started we were ignorant but now that we understand it*, *we have trust in you (NHRC) and we know you are helping us* ***(IDI with 48 year old Mother)*.**

*“There were so many diseases before NHRC came*. *The drugs that they have been giving to children have helped to drive away many diseases*. *Look at this my child*, *it was measles that has made him so*, *at the time he was born*, *there was no NHRC*. *NHRC has come to drive away measles and everything…*.*I have seen that they have done very well and I praise them for that*. *They have done a lot for my child…*. *My mother had nine children and measles killed seven of them…*. *That child had measles when we were down south; the others who were here (study districts) have not suffered from measles”* ***(IDI-39 year old Mother)*.**

Most parents also held the view that clinical trials had helped to reduce the occurrence of diseases such as malaria, cerebrospinal meningitis, and diarrhea in the communities. Parents attributed the reduction to the health education activities that health workers undertook as part of the conduct of clinical trials.

*“Yeah I think it has helped to reduce certain diseases though I am not a “yezura tintonnu” (health worker) but in my view I think malaria and other diseases have been reduced and all is because of the health education we get from health workers when they take us to be part of these studies*. *Their work is good*, *they are really helping us a lot especially our children”* ***(IDI-44 year old Mother)*.**

### Quality of Medical Care

Majority of the parents in this study perceived the better quality of care offered clinical trial participants to bethe main reason why clinical trials were acceptable to them. A 41 year old mother whose child was recruited into clinical trial had this to say:

*“My child had good health because when the child took part in that study (rectal artesunate trial)*, *he has never had that type of sickness since then so because of that I have seen that it is beneficial to me*, *yes the drug is good” (****IDI- 41 year old Mother)*.**

They perceived that quality of care was better in the clinical trial than in the routine care. They mentioned that trial participants were given better attention by study nurses and doctors. They were also followed-up at home by trial staff.

*“When you are referred to the health facility by the NHRC people*, *they would take good care of you quickly than when you go there alone for routine care*. *I was having a child who was part of one year study and when the child was sick*, *I took him there (referring to the health facility) and when I got there the study workers took the child quickly and sent him to the doctor for treatment*. *When the study was over and the child was not part of it again*, *we went to the hospital and we had to go and join the queue before we could see the doctor unlike the first one where they just took the child straight from me to the doctor for examination*. *So to me*, *if you are part of a study the care is faster and better than the routine care”* ***(IDI- 38 year old Mother)*.**

Few of the parents were of the view that clinical trials were conducted to help the poor to take care of their children. They added that they did not pay for their hospital bills when they were recruited into these trials and this helped them to be able to take care of the other needs of their children.

### Impact of Clinical Trials on Health Seeking Behavior

Parents who took part in this study reported that the participation of their children in clinical trials had positively influenced their health seeking behavior. They noted that clinical trials had helped them and their family members to patronize hospital treatment. They recalled that in years past, they depended largely on traditional medicine to cure illnesses. They said that the conduct of trial studies had improved their knowledge on the benefits of seeking early treatment at the health facility. They said that health facilities have the capacity to administer infusions (blood and drips) to patients when required as compared to traditional treatment. A 47 year old mother shared her view this way on the issue:

*“We used to patronize herbal treatment but because of our involvement in these studies*, *we now know that if you fall sick*, *the first place to go is the health facility because the herbalist may treat you but cannot do blood transfusion if the need arises*. *I am extremely happy that it has helped me*. *They said if someone is sick*, *I should do well to take the person to hospital and I have seen the benefits in doing that*. *NHRC has shown us the way because in the past if someone was sick*, *we used not to take the person to the hospital but rather apply various kinds of herbs on the body but now hospital first before”…****(IDI with 47 year old Mother)***

### Impact of Clinical Trial on Patient Referrals

Almost all the parents who took part in this study reported that the conduct of clinical trials had improved on patient referral from the community level to the health facility because of availability of transportation. They said that community-based staff of clinical trials used their motorbikes or vehicles to transport patients with severe health conditions to the health facilities. Some of them particularly mentioned the “Bajer” (tricycle) that was used in the rectal artesunate trial.

*“Yes for that one when you people were working with us if only they call your people*, *they would come and pick the person to the hospital either with a car or motor*. *That study that my child was part of*, *they had this small car with “three legs” (three-wheel) that they were using to carry the people to the health facility and I wished that type of small car was still there to help people”* ***(IDI-40 year old Mother)*.**

## Perceived Disadvantages of Clinical Trials

### Perceptions on Exposure to Risks during Clinical Trials

Few of the parents however, highlighted some issues they did not like when their children took part in these trials. They held the view that taking of blood samples from sick children was painful and made the children to cry. They also expressed concern about how staff engaged in clinical trials still went ahead to draw blood samples from children who already looked weak and pale. A 39 year old mother had this to say on the issue:

*Q*: *What problems or risks did your child get in the trial study you took part?*

*R*: *You always think that*, *“oh*, *this child is sick and yet they are taking his/her blood; his blood is not much” but what can you do?*….***(IDI-39 year old Mother)*.**

Adverse effects were also reported by some of the parents. They opined concern about possible suffer adverse effects of trial drugs and the possibility it could cause disabilities. One parent explained that her child had developed speech problems (stammering) a situation she believed might have been as a result of enrolling the child in a clinical trial.

*“Yes but what I have to say small is that my child who was part of the study*, *at the moment the child cannot talk well (child stammering) and I don’t know why whether it is because the child was given that drug or what?”* ***(IDI-34 year old Mother)***

Another parent was of the opinion that individuals involved in clinical trials suffered psychological trauma as researchers reveal findings of studies which hitherto were unknown to them and other community members.

*“hmm we have advantages and disadvantages…this can also bring about panic to the people around when they get to know that there is a particular disease or sickness that is within the area mean while they people don’t know and that can make them feel bad because they did not know that there is something like that within them”* ***(IDI- with 46 year old father)*.**

One father expressed the view that clinical trial participants were being used as guinea pigs. He explained that these trials drugs could work well for some participants and not so well other participants.

*“Yes there are risks because you test and the results are positive*, *you test and the results are negative and so there is a risk there but you cannot actually succeed in life without taking risks*. *Let’s say that this drug is taken orally*, *it can burn your triode when you are taking it and the intestines can be destroyed and you can be sorry and these are some of the risks involved*. *So my view about these trials is that people are being used as guinea pigs because sometimes it may come out well for some participants and another time things may not work well for other participants”(IDI with 48 year old father)*

### Perceptions on Drawing and the Use of Samples

Few parents were not happy about the taking of samples from their children. They appeared to express a sense of helplessness as they perceived they had no choice other than to allow researchers to take such samples from their children. They added that this was because the children were sick and needed help, and they had consented to the procedures of trials. Some parents feared that blood taken from their children could get tested for HIV. These were expressed with a mixed sense of mistrust and anxiety. Further to the issue of mistrust was the suspicious expressed by few participants that blood samples were sold to local and international partners. A number of parents were particularly worried that researchers were not coming back to tell them what they found in the samples they took from their children.

*R*: *The blood sample I must say that though you normally take it as part of these studies but at the end of the day we don’t normally know what you use the blood for whether you take it and sell or not we don’t know*. *We think that you normally use it for other things rather than what you said you were going to take it for and that had been the problem with the blood taking*.

*Q*: *Is that the way people think in your community?*

*R*: *Yes even me*, *and that is why certain times I may not want people to take my blood samples because you may go and use it to test for HIV*. *So sometimes we are not happy about it* ***(IDI-48 year old father)*.**

## Discussion

In this study we have found that parents’ perception of clinical trials is mixed and based on reasonably-founded rationalization of the actions of the researchers and the research centre. There is impressive appreciation of the role of clinical trials in developing new and safety medicines. This is possibly a result of the fact that the population in the study area was familiar with the conduct of clinical trials and must have heard the basis of the conduct of clinical trials. The findings are however consistent with the findings of similar studies in both high and low income countries [5, 11, 12].

While those who exhibited knowledge of clinical trials did so impressively, few of them were not able to mention the reasons for the conduct of clinical trials. This could be attributed to poor sensitization and education prior to trial implementation. It might also be blamed on poor consenting before parents take a decision to allow their children to participate in these new drug trials. It might also be as a result of parents’ eagerness to get medical attention for their sick children which compels them to accept participation of their children without knowing why these trial studies are conducted. The finding in this regard is consistent with the findings from similar studies in other parts of Sub-Saharan Africa [8, 10, 20].

The study has shown how overall collateral benefit accrues for the conduct of clinical trials when the work of a research centre is seen to result in reduction in morbidity and mortality. Parents in this study perceived a link between the work of the research centre and decline in the burden of disease in the districts. The perception of parents in this regard coincides with the findings of epidemiological studies in the districts that have demonstrated substantial reduction in child and adult mortalities. [21, 22]. While acknowledging these as objectively verifiable, it needed to be recognized that community members tended to exaggerate the position. For instance, while the research centre has not undertaken any major work in the control of measles, measles was among diseases often mentioned by respondents. Ghana has overall experienced substantial reduction in the incidence of immunizable diseases.

The study revealed that people who take part in trial studies have access to quality health care. They also have access to drugs, free and prompt access to medical care. These findings coincide with those in other studies [5, 23]. It is clear that parents placed a high premium on the quality of care their children received when they were recruited into these trials and this influenced their favorable perception of clinical trials. The study has thus demonstrated the irony that unavoidable improvement in the quality of care offered clinical trial participants leads to a preference over routine care. This well-founded basis, on the other hand potentially undermines the integrity of the informed consent procedure. There is some degree of compulsion to enroll in clinical trials that is imposed by systemic failings of the routine health delivery system.

The health-seeking behavior of trial participants was also reported to have improved tremendously as a result of the conduct of clinical trials and other activities of the research centre. Of particular mentioned was the community-based health planning and services concept where nurses are stationed at the community level to offer basic health care services including health education to people. The CHPS concept was carried out by the NHRC which is now a policy in Ghana [15, 16]. Because of the nearness of these health facilities to the people couple with the health education and the activities of clinical trials, people are usually encouraged to visit the health facility for health care services. Earlier studies reported that trials participants had access to health education and scientific knowledge as compared to non-trials participants [9, 10]. It is not therefore surprising for parents to associate the availability of health facilities and improved medical care with their participation in clinical trials.

Clinical trial participants in this setting are also exposed to some disadvantages or risks for their involvement in clinical trials. A particular concern is the crying of children during samples taking and possible side effects such as burning of triode and possible death. It has been reported that uncertainty in the efficacy of new drugs and the side effects new drugs may have on the liver and kidney are source of concern to trial participants [8, 9, 10]. It is therefore important that clinical trials in this and similar settings ensure full disclosure of information including anticipated risks, benefits and trial procedures [9]. Most of the parents who took part in this study however, expressed strong belief in the efficacy of study drugs used in this setting. Apparently, this trust is based on long history of good research conducted by the NHRC over the years in the communities.

The drawing of blood, processing and analysis are inevitable accompaniment of clinical trials. Studies have shown that communities or study participants may hold views that are inconsistent with the motives and principles of the conduct of clinical trials [11]. Misconceptions’ regarding the use of samples affected recruitment and retention of study participants in clinical trials [11, 12]. Our findings revealed varied opinions on the drawing and use of blood samples. The dominant opinion by parents that samples are drawn to establish the causes of illness for appropriate treatment might be explained by participant’s exposure to research trials. Thus, future clinical research operations in this setting will most likely attract community support and participation. However, attention should be paid to isolated misconceptions regarding the taking of blood samples during clinical trial studies for sale or for testing HIV. These views were apparently fueled by a perceived weak community engagement and poor communication on findings made on blood samples. This therefore raises questions on the actual use of blood samples by trial participants.

Traditionally, the aims and objectives, study design and procedures, participant’s rights and procedures are usually spelt out in participant’s informed consent forms for completion. Similarly, durbars are usually organized (community meeting of chiefs and people) where aggregate results from trials are disseminated. Poor organization and low community patronage of these meetings would imply only few community members receive information on the results of clinical trials. It is also possible that research trials may have failed to conduct disseminations on findings of their studies. Therefore, strengthening community involvement prior to trials implementation and effective community based disseminations would thus be key in dispelling negative perceptions on the use of samples taking during clinical trials. This will help in sustaining trials participants and other community members’ interest and support in the conduct of clinical trials [24].

## Conclusion

Generally, there is positive perception on the conduct of clinical trials in the Kassena-Nankana districts. It is therefore unlikely that the community members will reject trials undertaken under the aegis of the research centre in the study area. The reported negative attributes cannot however be ignored either. Misconceptions abound about the use to which blood samples are put and the unknown effects of new drugs must be given serious attention. The process of community engagement should be continuous and never compromised in the pursuit of an enabling community attitude towards clinical trials in this and other districts in sub-Saharan Africa.
